# Notes on the Use of Nitro-Glycerine in the Treatment of Heart Disease

**Published:** 1882-04

**Authors:** W. E. Green

**Affiliations:** Isle of Wight


NOTES ON THE USE OF NITRO-GLYCERINE IN THE TREATMENT OF HEART DISEASE. By W. E. GREEN, M.R.C.S., Isle of Wight.

I propose to bring before you certain results obtained by me in the treatment of heart disease with a somewhat recent remedy, viz., nitro-glycerine. This is a most potent remedy, and I believe I am not overstating its merits when I say that it deserves to rank only second to digitalis in the treatment of disease of the heart.

The action of the drug, which was first noticed by Field, of Brighton, has been experimented upon by various observers, chief among whom may be mentioned Hering, Demme, Alders, Onsum, Brady, Pelika, Thorowgood, Eulenberg, and Weber. In 1876 Dr. Lauder Brunton wrote a valuable paper in the St. Bartholomew's Hospital Reports, detailing its peculiar physiological action upon animals, as tested in the pharmacological laboratory of that hospital. It is to Dr. William Murrell, however, that the medical profession is chiefly indebted for introducing it as being of great value in the treatment of certain diseases. From a consideration of the physiological action of this substance, and especially from the similarity existing between its general action and that of nitrite of amyl, as set forth by Dr. L. Brunton, Dr. Murrell was led to infer that it would probably be of service in the treatment of angina pectoris, and his result may be seen by a reference to his valuable paper in the *Lancet* of the early part of the year 1879.

I will briefly detail its mode of action and the preparation usually employed.

The solution is the form most usually used, and this is a 1 per cent. solution in spirits of wine. One minim is the usual dose to commence with, but in some cases even less may be given with advantage. It can either be taken in water or one drop may be placed upon the tongue. The solution is almost tasteless, but within three minutes of being taken it begins to exert its peculiar physiological properties. It paralyzes the vaso-motor nerves, and so dilates the blood-vessels; the face flushes, the temples throb, the pulse becomes dicrotic and much quickened; in some cases the head aches most violently, but in others only a sense of fullness and pain across the forehead is experienced, which lessens with each recurring dose, until ultimately no unpleasant effect but simply a warming sensation all over the body, is produced.

A feeling of nausea, or even sickness, is often caused by the earlier doses. The quantity may be gradually increased until fifteen or twenty minims every four hours are given, but I have never found it necessary to administer such heroic doses. It is never wise to give more than one minim at first, for even this small quantity has produced most serious symptoms in certain individuals. The patient has fainted, and has become almost collapsed, but I am not aware that it has ever been followed by a fatal result. I have never myself seen these alarming symptoms, but in two cases have found unpleasant symptoms to succeed a first dose, both cases occurring in hysterical women. The medicine, in one case, was taken without my being consulted (the patient having seen it produce good effects in her father's case); in the other, I prescribed it for certain nervous symptoms, but, at that time, not having had so much experience in its use, I did not like to persevere. The physiological effect of nitro-glycerine is not so rapidly produced as is that of nitrite of amyl, but it continues from four to six or even eight hours, after which time it is often advisable to repeat it.

I have never found it produce unpleasant effects in any case where its use was plainly indicated, and each day's experience more clearly shows the cases likely to be benefited by it.

While useful in almost all cases of heart disease, I believe those in which it will be found of the greatest benefit are, first, angina pectoris, and second, weak, dilated, and fatty heart. In angina it prevents an attack by keeping the blood-vessels in a constantly dilated condition, and thus prevents the backward pressure of blood upon the heart, which is probably the cause of the agonizing pain of angina.

In weak, dilated hearts it gives relief by reducing arterial tension, and thus lessening the amount of work the heart has to do. The heart, consequently, gains in power by the rest so given to it. As a rule, digitalis does not agree in these cases; but, if thought necessary, it may be given with increased advantage in conjunction with this drug. In several cases of dilated heart, with small, weak, quick pulse, I have seen the beats not only increased in power, but much reduced in frequency, after taking nitro-glycerine for a few days, thus plainly showing that the heart had been relieved of much of its embarrassment, and as a consequence had gained in power. I have used this drug largely during more than two years, and each week my appreciation of its value as a remedy for this classs of cases increases. There are numerous other affections in which it will prove of value, but such do not come within the scope of my present paper. Bearing in mind its physiological action, it will be easy for you to select the cases in which its use is indicated. (The author relates here half a dozen remarkable cases, and adds:) I could relate numerous other cases showing the value of this remedy in heart disease. The cases cited, however, are sufficient to prove that in this new agent we have a very powerful measure in diseases of the heart. It is a remedy, moreover, which is not only palliative, but in many cases actually curative, and I think I may lay claim to having discovered its application to a much larger and more common class of casses than those originally laid down by its introducer, Dr. Murrell.—*The Practitioner*.

